# Inferences for the Lead Time in Breast Cancer Screening Trials under a Stable Disease Model

**DOI:** 10.3390/cancers3022131

**Published:** 2011-04-26

**Authors:** Justin Shows, Dongfeng Wu

**Affiliations:** 1 Department of Mathematics and Statistics, Mississippi State University, PO Box MA, Mississippi State, MS 39762, USA; 2 Department of Bioinformatics and Biostatistics, University of Louisville, Louisville, KY, USA; E-Mail: dongfeng.wu@louisville.edu

**Keywords:** breast cancer, early detection, sensitivity, sojourn time, periodic screening, lead time

## Abstract

We estimated the effects on the lead time for women participating in a long-term breast cancer screening program when the screening sensitivities and disease progression are independent of age. The lead time, or time by which a diagnosis is advanced by screening, is one of the major concerns of any cancer screening program, which we consider to include both a mammogram and physical examination. Using estimates of test sensitivities and mean sojourn times previously calculated by other authors from observed data, we estimated properties of the lead time. We utilized the model for the lead time derived by other authors and ran simulations for different screening program designs, concentrating on screening interval lengths of 0.5 years, 1 year, 1.5 years, and 2 years. These estimates were based on a long-term screening program from age 50 to 80. For each six-month decrease in screening interval length, we estimated the percent increase in mean lead time, as well as the percent increase in the proportion of clinical patients who will have their cancer detected at a screening exam.

## 1. Introduction

Breast cancer is the most common form of cancer among women in the United States and the second leading cause of cancer deaths among women [1]. If breast cancer can be diagnosed early, then treatment can prolong the life of a patient or even provide a cure. Early detection improves the chance of survival as it allows treatment to begin before the cancer reaches an advanced stage [2,3]. Therefore, the ability to detect breast cancer early plays a crucial role in determining the effectiveness of a screening program [4]. Obviously, more screenings at shorter intervals would probably improve the chance of early detection, but practical factors such as cost, time, healthcare resources, and possible adverse physical effects, must be considered [5], including the possible increase in false positive results and over-diagnosis. We have not yet considered the impacts of these factors on the screening program, which will be an area for future research. In order to design an efficient screening program, some of the most important elements to be considered are the ages at the initial and final exams and the frequency of subsequent exams [6]. A stable model, which is independent of age, such as that considered by Baker [7] will be assumed. The results may lead to inferences that can be used to investigate a model that depends on age. We will concentrate on the frequency of the exams, considering equal time intervals between screenings. An optimal screening program will provide an early detection for as many people as possible, while still taking into account the adverse effects of the screenings. If a patient's cancer is detected early, then the screening program has the potential to benefit that patient if she is able to receive effective treatment at the early stage. If not, then the program provides no benefit to the patient, since the treatment will begin at the same time as if no exams had been given.

Two major indicators of the efficacy of any screening program are the lead time, which is the length of time the treatment can be advanced by screening exams, and the sensitivity of the exam [8]. Wu *et al.* [9,10] built a probability model for the lead time and then used it to derive the exact probability distribution for the lead time. We will use this distribution to make inferences about the lead time, such as its mean and the proportion of women who will potentially not benefit from the program by early detection. The effects of exam frequency (interval length) on these properties can also be examined. Simulations will be used to estimate these effects. Based on our results, doctors and patients can weigh risk and cost factors against the increase in benefits of a screening program.

## 2. Patients and Methods

### 2.1. Patients

The data used in obtaining estimates was collected in three controlled studies, the Health Insurance Plan of Greater New York (HIP), the United Kingdom Trial of the Early Detection of Breast Cancer (Edinburgh), and the Canadian National Breast Screening Studies (CNBS).

The HIP study began in 1963 with 62,000 initially asymptomatic women with ages ranging from 40 to 64. The women were randomly divided into two equally-sized groups. The study group received a clinical physical examination and a mammogram at the initial screening. The screenings were repeated annually for the next three years. The women in the control group followed their normal health care routines [11].

The Edinburgh study began in 1976 with 45,130 women aged 45 to 64. None of the women had ever been diagnosed with breast cancer. Screenings were conducted annually for subjects randomly assigned to the treatment group. In the first, third, and fifth screenings, both a physical examination and a mammogram were given. A physical examination only was given in the second, fourth, and sixth screenings [12].

The CNBS study, which began in 1980, consisted of 50,340 women aged 40–49 (CNBS1) and 39,405 women aged 50–59 (CNBS2). None of the women had a history of breast cancer or had received a mammogram during the previous year. The women were randomly assigned to the control group or the study group. The study group of 40–49 year-olds received an initial screening of a mammogram and physical examination, with four annual re-screenings. The control group of 40–49 year-olds received only an initial physical examination. The 50–59 year-old study group received an initial screening of a mammogram with four annual re-screenings. The 50–59 year-old control group received an initial physical examination with four annual physical examinations [13,14].

### 2.2. Methods

**Scheme 1.**
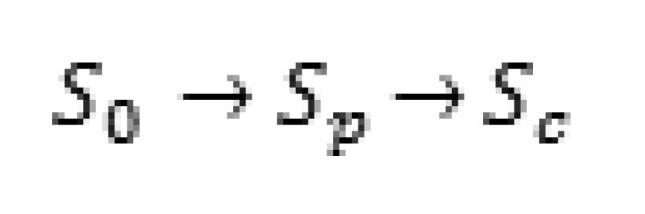
Disease progression.

For the lead time to exist, a woman must develop cancer at some time during the screening program. Our model will be for all women who develop breast cancer during the program, including those whose cancer is detected at a screening and those whose cancer is not detected. To build our model for the lead time, we must assume that the disease can be in three stochastic states and progresses in the following manner, as defined by Zelen and Feinlab [15], illustrated in Scheme 1.

where S_0_ is the disease-free state, where no disease is present, S_p_ is the preclinical state, where the disease is present but physical symptoms have not yet appeared. In this stage, an individual is unaware of the disease, but it can be detected by a screening exam. S_c_ is the clinical state in which physical symptoms are present. In this state, the cancer could be detected by a routine physical or even by the patient herself, so any special screening exam would be unnecessary. If a patient develops clinical cancer which was not detected in the preclinical stage before a scheduled exam, then the cancer will be found by the examiners before the next screening is given. Therefore, we would assume that all individuals participating in a screening program are either in S_0_ or S_p_ at the time of the initial screening. These subjects enter the screening program at age t_0_, which is typically between 40 and 64, inclusive. Throughout this paper, we will assume that a screening exam consists of both a physical examination and a mammogram. Chen *et al.* [12] considered the effects of a physical examination, a mammogram, and both, and we will use their estimates from the cohort receiving both elements.

For our model, we will need the transition rate *w* which is the yearly probability of an individual making a transition from S_0_ to S_p_. The sojourn time, or time spent in S_p_, will have mean λ. The age at which the last screening is offered is denoted by *T*, for which 80 could be considered a reasonable upper bound. The sensitivity of each screening will be denoted by β. The sensitivity is the probability that cancer is detected in a patient if that patient is truly in the preclinical stage. We will not consider the probability of a false positive result at this time. The lead time, the time by which the diagnosis and treatment are advanced by screening, is denoted by *L.*

The goal of any screening program should be to detect the cancer while individuals are in the preclinical state S_p_, so that treatment may begin earlier in the hope that the mortality rate will be reduced [3]. If S_p_ begins at time T_1_ and S_c_ begins at time T_2_, then the sojourn time will be T_2_-T_1_, where T_1_ is unobservable. We would hope to detect the cancer at some time *t* in the interval (T_1_,T_2_). If cancer is detected by a screening exam at time *t*, then the lead time is T_2_-*t*. If cancer is not detected before T_2_, then the lead time is zero. The individuals with positive lead times may potentially benefit from the screening exams as their treatment can begin at an earlier stage, while the individuals with lead times of zero will not. The lead time is crucial not only to the effectiveness of a screening program, but to an individual's health as well. A longer lead time will allow a cancer patient to begin treatment earlier, thereby increasing her chances of survival [12]. In order to investigate the properties of the lead time, we must use estimates for the parameters previously mentioned. As stated before, the time at which the preclinical stage begins is unobservable. This means that a patient's sojourn time cannot be exactly determined either. Since there is no completely error-proof method in determining the true disease status of an individual (except possibly a biopsy, which is medically and economically unfeasible to give to every woman at every screening), the true sensitivity of a screening is not known.

The model of the progression of the disease and screening program can be considered either stable or unstable. With a stable model, the transition rate and sensitivity are independent of age. The transition rate and sensitivity are age-dependent under the unstable model. For estimates of the sensitivity, a screening exam is assumed to consist of both a physical examination and a mammogram. Under the stable model, the transition rate *w* and the sensitivity β are constants, and the sojourn time is exponentially distributed with a mean of λ. This allows the model to take a much simpler form, but largely ignores the effects of age on disease progression. Previous work [16-19] has claimed that women in their 40s tend to have a shorter sojourn time than older women, which would influence the sensitivity and therefore the mortality rate. However, the age-dependent model takes a much more complicated form, and we will only consider the stable model and apply our simulation results to patients who begin a screening program at age 50.

According to the National Cancer Institute's SEER database [20], 12.15% of women are diagnosed with breast cancer, so the probability that a woman develops breast cancer (enters the preclinical stage) at some point in her life is 0.1215 or higher. Since this represents a woman's lifetime risk of developing breast cancer, we know that this probability is at least 0.1215, and a reasonable upper bound of 0.20 can be chosen for *w*. If we assume that the transition can happen between the ages of 30 and 80, then a period of 50 years is considered and an estimate for the yearly transition rate is *w* = 0.20/50 = 0.004. Under the stable model, the distribution of the lead time is independent of the transition rate. It depends only on the sojourn time, sensitivity, and interval length between exams. Based on observed data from each of the studies, Chen *et al.* [12] found the maximum likelihood estimators for β and λ. The estimates are given in Table 1, along with their standard deviations in parentheses.

## 3. Results

Under the unstable model, Wu *et al.* [9,10] derive a probability distribution for the lead time as a mixture with a point mass at 0 and a piecewise continuous positive random variable. This model extends that of Prorok [5] to include interval cases as well as those who are detected on exams. Interval cases (previously undiagnosed women who enter the clinical state between screening exams) will not be detected by screening and will have a lead time of 0, while women who are detected while still in the pre-clinical state will have a positive lead time. As a result, the probability model has a point mass at zero with a piecewise continuous probability density function (pdf) with positive support. Let *L* be the lead time for those detected during the screening program, and *D* be a binary random variable that takes the value 1 if clinical disease develops before *T*, and 0 if it does not. The proportion of clinical cases that will not be detected during the screening program is then the conditional probability:

Equation 1. $$P(L=0|D=1)=\frac{P(L=0,D=1)}{P(D=1)}$$

This represents the proportion of women who will enter S_c_ before T, but will not be detected at any time during the screening program. The probability *P*(*L* = 0, *D* = 1) is the joint probability that an individual will both develop clinical disease before *T*, and that it will not be detected by any of the screening exams (*i.e.*, be an interval case).

We will consider a screening program that calls for *K* exams given at ages t_0_ < t_1_ < … < t_k-1_, with an equal screening length Δ between exams, so that t_i_ = t_0_ + *i*Δ for *I* = 1,2,…,*K*-1. We will define *I*_K,i_ as the probability that an individual becomes an interval case in the *i*th interval (t_i−1_,t_i_), where t_−1_ = 0 and t_K_ = *T*. The lead time is 0 if and only if an individual is an interval case. So, we can write the total probability that an individual will be an interval case as the sum of the probabilities of becoming an interval case in each of the intervals:

Equation 2. $$P(L=0,D=1)=I_{K,1}+I_{K,2}+\cdots +I_{K,K}$$

The derivations of Wu *et al.* [9,10] represent these probabilities as integrals involving a continuous age-dependent transition rate function and an unspecified sojourn time distribution. The derivations of the equations were given in great detail in these papers, so we refer the reader to the works of Wu *et al.* [9,10]. Under the stable model, the transition rate is constant, and the sojourn time has an exponential distribution with mean λ. Because of this, the integrals given by Wu *et al.* [9,10] can be evaluated and simplified to the form given in Equation 3, and are functions of only the transition rate, mean sojourn time, sensitivity, and time interval length. Each term in Equation 2 can be written as:

Equation 3. $$I_{K,j}=w\lambda (1-\beta )\;(e^{\frac{-\Delta (j-1)}{\lambda }}-e^{\frac{-t_{j-1}}{\lambda }}-e^{\frac{-\Delta_{j}}{\lambda }}+e^{\frac{-t_{j}}{\Delta }})+w\lambda \sum_{i=1}^{j-1}\;\left(e^{\frac{-\Delta (j-i-1)}{\lambda }}-2e^{\frac{-\Delta (j-i)}{\lambda }}+e^{\frac{-\Delta (j-i+1)}{\lambda }}\right)\;{\left(1-\beta \right)}^{j-i}+w\Delta -w\lambda +w\lambda e^{\frac{-\Delta }{\lambda }}$$

for j = 1,2,…,*K*, under the stable model. To find the probability that an individual will enter the clinical stage before *T* under the stable model, we can apply the derivations of Wu *et al.* [9,10] to obtain.

Equation 4. $$P(D=1)=w(T-t_{0})+w\lambda (e^{-T/\lambda }-e^{-t_{\text{o}}/\lambda })$$

This equation is a simplification, under the stable model, of those derived in detail by Wu *et al.* [9,10]. To obtain the conditional probability P(L = 0|D = 1) given in Equation 1, we take the ratio of P(L = 0, D = 1) and *P*(*D* = 1). From this, we can see that the conditional probability given in Equation 1 is independent of *w*, and depends only on the sensitivity of the screening β, the mean sojourn time λ, and the interval length Δ.

Using this model and the MLEs from Chen *et al.* [12], we can estimate the proportion of clinical patients who will not potentially benefit from early detection in the screening program, and the mean lead time for all clinical patients. The proportion of clinical cases that receive no benefit from early detection is denoted by *P*, which is equal to *P* (L = 0|*D* =1*)*. The proportion of clinical patients who potentially benefit from the program by having an early detection is then *Q* = 1 − *P*, the probability that the lead time is positive. Obviously, smaller values of *P* and larger values of *Q* indicate a better screening program for patients who will eventually enter the clinical stage. We will focus on the time interval between exams, denoted by Δ. Intervals of 0.5 years, 1 year, 1.5 years, and 2 years are considered. For purposes of comparison, we will examine the case of women who have their first screening at age 50 and the last at age 80. Table 2 gives the resulting estimates for *P*, *Q*, mean lead time (in years), standard deviation of the lead time (SD), and the ratio of mean lead time to mean sojourn time.

For these estimates to be useful to patients and doctors, we will further quantify the benefits of decreasing the time interval between exams. If exams are given more frequently, we expect the mean lead time to increase and the proportion of clinical cases having an early detection to increase. We will use our estimates to calculate the percent increases in both mean lead time and *Q* for each six-month decrease in time interval length. Table 3 gives estimates for percent increases in mean lead time and *Q* for each study and each 0.5-year decrease in screening interval length. For instance, according to the results from the HIP data, if a woman is being screened yearly and considers having a screening every 0.5 years, we estimate that, if she does eventually enter the clinical state, her chances of being diagnosed in the clinical state will increase by 15.88%, and that the expected lead time will increase by 17.11% if the change is made.

Assuming that the sensitivity, transition probability, and sojourn time are age-dependent, Wu *et al.* [10] applied their probability model to the HIP data using Bayesian posterior samples. They also considered a periodic screening program for women aged 50–80 years given equally-spaced examinations. Using the HIP data, they estimated *P*, *Q*, mean lead time, and standard deviation. Their results are given in Table 4. Our estimate for *P* is much larger if screenings are given every six months but, as the time interval between exams increases, the estimates tend to agree. However, our estimates for the mean lead time are much more optimistic.

## 4. Conclusions

In this paper, we have examined the “long-term” effects of screening programs. Many previous studies cited here have focused on at most six screenings, but we are using the parameter estimates for β and λ to apply to a 30 year analysis for a woman having equally-spaced screenings from age 50 to 80. The distribution for the lead time is for the whole diseased group, including both the screening-detected cases and the interval incidence cases that are not detected by screening. This allowed us to find estimates for *P* and *Q.* The estimates for *P* indicate that the proportion of breast cancer patients who do not benefit from a screening program increases as the time between screenings increases. Also, the mean lead time and the proportion of women who will have an early detection (*Q*) both increase when the time interval length decreases. While more frequent screenings will probably benefit more people, the increase in benefit must be weighed against the increases in cost of additional screenings, time of investigating false positive test results, and possible adverse physical effects due to screening. Doctors and patients should first look at additional risk factors, such as family history of breast cancer and overall health before examining the benefits of more frequent screenings. Once these factors have been assessed, the percent increases in mean lead time and *Q* can be used to determine whether the extra costs are justified by the benefits. They should be warned, however, that none of the estimates presented take age into account. Several authors [16-19] have shown that, especially for younger women, sensitivity tends to be lower and sojourn time shorter.

Since each of these studies resulted in different MLEs for β and λ, the estimates for *P*, *Q*, Mean, and Ratio also differ. This could be related to the mean sojourn time λ. Since the lead time cannot be longer than the sojourn time, we expect the mean lead time to increase if the mean sojourn time is larger. The number of women who could potentially benefit from the screenings by having an early detection would then tend to be greater. For instance, in the Edinburgh study, the estimate for the mean sojourn time is the largest of all the studies, and the resulting estimates for *Q* are also the largest. Since the estimates for the mean lead time increase as the mean sojourn time increases, we suggest using the ratio of the mean lead time to mean sojourn time as an indicator of relative effectiveness. This is because bias in the estimation of the sojourn time will cause bias in the estimation of the lead time.

For future research, we will consider the unstable model, which is more realistic for the screening trials. We also plan to consider false positive results and over-diagnosis, costs, and adverse physical effects due to screening, and the impact these factors have on the screening program. Individual risk factors such as family history and environmental effects will also be evaluated.

## Figures and Tables

**Table 1.** Parameter estimates for the four screening trials.

**Study**	**β (Sensitivity)**	**λ (Mean Lead Time)**
HIP	0.70 (0.20)	2.50 (1.20)
Edinburgh	0.78 (0.04)	4.30 (0.37)
CNBS1	0.91 (0.18)	1.90 (1.20)
CNBS2	0.82 (0.12)	3.10 (0.94)

**Table 2.** Lead time estimates for the four screening trials.

**Study**	**Δ (Interval Length)**	***P* (Proportion of clinical cases with lead time of 0)**	***Q* (Proportion of clinical cases with positive lead time)**	**Mean Lead Time**	**Standard Deviation of Lead Time**	**Ratio of Mean Lead Time to Mean Sojourn Time**
HIP	0.5	0.168	0.832	1.916	2.236	0.767
	1	0.282	0.718	1.636	2.145	0.654
	1.5	0.375	0.625	1.403	2.079	0.561
	2	0.450	0.550	1.307	2.042	0.523
Edinburgh	0.5	0.110	0.890	3.384	3.653	0.787
	1	0.180	0.820	3.188	3.599	0.741
	1.5	0.242	0.758	2.755	3.384	0.641
	2	0.297	0.703	2.641	3.440	0.614
CNBS1	0.5	0.145	0.855	1.595	1.817	0.840
	1	0.257	0.743	1.327	1.741	0.699
	1.5	0.348	0.652	1.162	1.632	0.612
	2	0.424	0.576	1.077	1.661	0.567
CNBS2	0.5	0.120	0.880	2.475	2.696	0.798
	1	0.206	0.794	2.129	2.563	0.687
	1.5	0.281	0.719	1.983	2.592	0.640
	2	0.345	0.655	1.794	2.559	0.579

**Table 3.** Benefits of decreasing screening interval length.

**Study**	**Change**	**% Increase in Mean Lead Time**	**% Increase in *Q***
HIP	1 to 0.5	17.11	15.88
	1.5 to 1	16.61	14.88
	2 to 1.5	7.35	13.64
Edinburgh	1 to 0.5	6.07	8.54
	1.5 to 1	15.72	8.18
	2 to 1.5	4.32	7.82
CNBS1	1 to 0.5	20.20	15.07
	1.5 to 1	14.20	13.96
	2 to 1.5	7.89	13.19
CNBS2	1 to 0.5	16.25	10.83
	1.5 to 1	7.36	10.43
	2 to 1.5	10.54	9.77

**Table 4.** Lead time estimates from Wu *et al.* [10].

**Δ**	***P***	***Q***	**Mean**	**SD**
0.5	0.090	0.910	1.418	2.111
1	0.234	0.766	1.168	2.040
1.5	0.365	0.635	0.988	1.969
2	0.467	0.533	0.856	1.901
